# An Epitome of the Newer Materia Medica

**Published:** 1887-02

**Authors:** 


**An Epitome of the Newer Materia Medica,** Standard Medicinal Products and Finer Pharmaceutical Specialties, introduced and manufactured by PARKE, DAVIS & CO., Detroit, Mich.

The profession is indebted to Messrs. Parke, Davis & Co., for the introduction into this country of many valuable remedies and also for much valuable information concerning them. The present volume contains certainly the best resumé on the subject of new remedies obtainable by the general practitioner. With the enterprise characteristic of the firm, they offer to send free to any physician applying for it, a paper-bound copy of the book. We would recommend all our readers to send for it.

